# Cognitive behavioral self-help interventions for individuals experiencing psychosis: a systematic review

**DOI:** 10.1017/S0033291724001545

**Published:** 2024-09

**Authors:** Emily Kruger, Laura Hall, Anton P. Martinez, Richard P. Bentall

**Affiliations:** Clinical and Applied Psychology Unit, Department of Psychology, University of Sheffield, Sheffield, UK

**Keywords:** CBT, cognitive-behavioral therapy, psychosis, self-help

## Abstract

Little is known about the effectiveness of cognitive behavioral therapy (CBT) specific self-help for psychosis, given that CBT is a highly recommended treatment for psychosis. Thus, research has grown regarding CBT-specific self-help for psychosis, warranting an overall review of the literature. A systematic literature review was conducted, following a published protocol which can be found at: https://www.crd.york.ac.uk/prospero/export_record_pdf.php. A search was conducted across Scopus, PubMed, PsycInfo, and Web of Science to identify relevant literature, exploring CBT-based self-help interventions for individuals experiencing psychosis. The PICO search strategy tool was used to generate search terms. A narrative synthesis was conducted of all papers, and papers were appraised for quality. Ten studies were included in the review. Seven papers found credible evidence to support the effectiveness of CBT-based self-help in reducing features of psychosis. Across the studies, common secondary outcomes included depression, overall psychological well-being, and daily functioning, all of which were also found to significantly improve following self-help intervention, as well as evidence to support its secondary benefit for depression, anxiety, overall well-being, and functioning. Due to methodological shortcomings, long-term outcomes are unclear.

## Introduction

Psychosis is a term used to characterize the ways in which people may perceive and process things differently from others, leading to difficulties with distinguishing what is real and what is not (Lieberman & First, 2018) and is usually accompanied by the presence of hallucinations (multi-sensory experiences with the absence of stimuli) and/or delusions (fixed false beliefs). Behavioral disturbances, and lack of insight into the pathologic nature of the experiences can also be present amongst some individuals (American Psychiatric Association, 2013).

Farhall, Greenwood, and Jackson (2007) first discussed how people with psychosis were capable of identifying their own coping behaviors to manage psychotic symptoms, demonstrating the potential natural ability that people with psychosis have in self-treating their experiences. Self-help approaches have been used for people with psychosis for many years (Snowdon, 1980). Self-help as an approach can be defined as resources made publicly available that focuses on helping people overcome psychological distress independently, in their own time (Scott, Webb, & Rowse, 2015).

Scott et al. (2015) conducted the first systematic review and meta-analysis into the effectiveness of self-help interventions for people with psychosis. Notably, they found that from 24 studies, self-help approaches had on average ‘small to medium’ effect sizes on overall psychotic-related symptoms, suggesting that self-help interventions have benefit for people with psychosis in reducing distressing symptoms. However, it was noted by the authors that only two out of the 24 studies within the review involved delivering a CBT-based self-help intervention, despite the fact that CBTp is highly recognized as a first-line psychological treatment for psychosis by NICE (2014). CBT-based self-help is known to typically involve patient-led resources focused on helping people recognize and change unhelpful thinking and behavioral patterns (Fenn & Byrne, 2013).

It was argued that this could be a potential gap in the research, and the authors suggested that future research should investigate CBT-specific self-help methods for people with psychotic presentations. Since this publication, the evidence for self-help approaches based on CBT principles for psychotic experiences has been growing, prompting the need for an updated review, considering the more recent research.

This current review aims to explore two key research questions: (1) what CBT-based self-help interventions have been developed for people experiencing psychosis? (2) what is the effectiveness of these interventions? In order to answer these questions, this systematic review will aim to identify quantitative research exploring forms of cognitive behavioral self-help interventions carried out with people experiencing psychosis. The quality of these studies will also be assessed.

## Method

The published protocol for this review was pre-registered on PROSPERO https://www.crd.york.ac.uk/prospero/export_record_pdf.php.

### Systematic review

**Figure 1.**
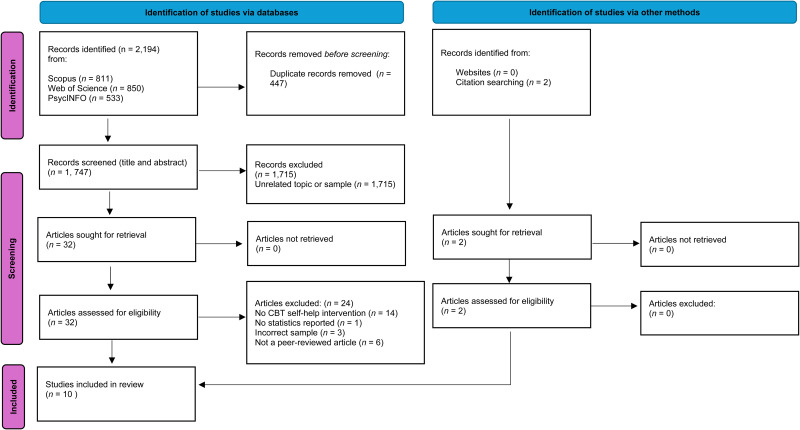
PRISMA flow diagram

A systematic search was conducted across four databases (Web of Science, Scopus, PubMed, and PsycInfo) in May 2023, in order to identify the literature investigating the effectiveness of CBT-based self-help interventions for psychosis. To select papers, the Preferred Reporting Items for Systematic Reviews and Meta-Analyses (PRISMA) guidance was followed in order to support the process of selecting the literature (Page et al., 2021). To improve rigor, the final completed PRISMA checklist is detailed in online Appendix A. Ethical approval was not required for this particular systematic review. The PRISMA diagram for this flow of the strategy can be found within Fig. 1. For additional clarification, the inclusion and exclusion criteria are shown in Table 1, and search terms for the review are shown in Table 2. Elements of the PICO search strategy tool (Richardson, Wilson, Nishikawa, & Hayward, 1995) were used to support the process of generating search terms for this review, and some search terms were derived from the review published by Scott et al. (2015). To improve the searching process, a consultation was also held with a university liaison librarian specializing in Psychology to check search terms before searching commenced. The support included the use of Boolean operators (AND and OR) to widen the search remit. Searches were limited from the year 1990 until present time, with the aim of including the relevant CBT self-help intervention papers reviewed by Scott et al. (2015) in order to provide a more rigorous update of the literature. The present review excluded papers which were not published in the English language. Grey literature was not included in order to maintain study quality (Pappas & Williams, 2011).

**Table 1.** Inclusion and exclusion criteria

Inclusion	Exclusion
– Quantitative studies	– Studies not published in English
– Peer-reviewed published research – Studies investigating the effectiveness of a form of self-help* intervention for psychosis based on CBT principles	– Grey literature – Unpublished literature
– Studies that recruited individuals experiencing symptoms relating to psychosis to receive a CBT self-help intervention – ‘Psychosis’ defined as a schizophrenia spectrum condition and other psychotic disorders as cited within the DSM-5	
– Studies measuring quantitative outcomes for psychosis symptoms	
– Studies measuring other quantitative outcomes on symptoms associated with psychotic experience e.g., quality of life, distress, or mood. – Quantitative statistics available	

*Note:* For the purpose of this review and in line with the previous review by Scott et al. (2015), a self-help intervention was defined in line with Bower, Richards, & Lovell's (2001) definition, whereby the intervention is conducted mainly independent of a mental health professional.

**Table 2.** Search terms

Construct	Search terms
Population	Psychosis OR psychotic OR schizophrenia OR ‘schizophrenia spectrum disorder’ OR ‘hearing voices’
Intervention	‘Self-help’ OR ‘guided self-help’ OR ‘self-monitoring’ OR ‘self-directed’ OR ‘minimal guidance’ OR ‘cognitive behavioral therapy self-help’ OR ‘CBT self-help’
Comparison	Not applicable
Outcome	Not applicable

### Study selection

Initial searches of the literature yielded 2194 papers across three databases. Articles were extracted to the Mendeley software program, and duplicates were removed, resulting in 1747 papers. Titles and abstracts of these papers were then screened by the first author and were checked against the necessary criteria, resulting in 32 articles meeting eligibility to be screened at full-text level. Articles were generally screened out at this stage if they did not include a CBT self-help intervention, a psychosis sample was not present, it was not a peer-reviewed article, or statistics were not reported. In addition to this, reference lists were finally searched, resulting in two additional papers. Thus, 10 papers were included within this current systematic review. During the screening process, an independent trainee clinical psychologist checked a proportion of the papers for screening at full-text level (15%) to ensure reliability. No discrepancies were reported during this process but would have been resolved through discussion. Study characteristics were extracted from the papers, which included author and date, origin of the study, participant demographics, type of cognitive behavioral self-help intervention, and outcome of the intervention and effect size. Searches were repeated in December 2023 whereby no additional articles were found.

### Quality appraisal

The quality of all papers was analyzed by the Effective Public Health Practice Project (EPHPP) Quality Assessment Tool for Quantitative Studies (Thomas, Ciliska, Dobbins, & Micucci, 2004; online Appendix B). The EPHPP is noted to be a well-established tool for assessing study quality, providing a standardized approach to assessing quality based on different quantifiable categories: selection bias, study design, confounders, blinding, data collection methods and withdrawals and dropouts. A trainee clinical psychologist, independent from the review, also rated a random selection of the papers (*n* = 50%), and any disagreements were planned to be resolved via discussion.

## Results

### Overall summary of studies

A total of 10 studies were included in the systematic review, whereby the previously stated PRISMA flow diagram clearly outlines the screening phases in Fig. 1. A ‘study characteristics’ summary can be located in Table 3 in alphabetic order, to provide information regarding study context and statistical results. Following the second-rater screening, 100% agreement was reached on whether papers should be included or excluded during full-text reviewing. The studies included in this review contained either cohort studies (*n* = 4) or randomized controlled trials (*n* = 6), in order to explore the effectiveness of a CBT self-help intervention. Regarding geographic location, three of the studies took place in the United Kingdom (UK) (Bucci et al., 2018; Hazell, Hayward, Cavanagh, Jones, & Strauss, 2018; Taylor, Strauss, & Cavanagh, 2021), three took place in the United States (US) (Gottlieb et al., 2017; Gottlieb, Romeo, Penn, Mueser, & Chiko, 2013; Granholm, Ben-Zeev, Link, Bradshaw, & Holden, 2012), two in Germany and/or Switzerland (Moritz et al., 2016; Westermann, Rüegg, Lüdtke, Moritz, & Berger, 2020). Finally, one study occurred in Portugal (Almeida, Couto, Marques, Queirós, & Martins, 2018) and one study was conducted in Canada (Naeem et al., 2016). In terms of service context, all studies took place in and recruited individuals from community settings. Thus, services ranged from early intervention in psychosis services (Bucci et al., 2018) to mental health rehabilitation centers (Almeida et al., 2018). The total number of participants included across the studies was 379. The sample included both male (*n* = 196), female (*n* = 182) and other (*n* = 1) participants. Mean participant average age ranged between 35.57 and 48.7 years. The mental health diagnosis of participants varied between studies, whereby three studies required participants to have a diagnosis of schizophrenia (Almeida et al., 2018; Gottlieb et al., 2017; Naeem et al., 2016), three studies required participants to have a diagnosis of either schizophrenia or schizoaffective disorder (Gottlieb et al., 2013; Granholm et al., 2012; Moritz et al., 2016), three studies required participants to have a diagnosis or experience of psychosis (Bucci et al., 2018; Hazell et al., 2018; Taylor et al., 2021) and, finally, one study had the requirement of a diagnosis of a schizophrenia spectrum disorder (Westermann et al., 2020).

**Table 3.** Study characteristics

Source and year	Title	Design	Country setting	Sample, mean age, gender, diagnosis	Intervention control group	Follow-up	Psychosis measures	Results	Other measures	Results
Almeida et al. (2018)	Mobile application for self-management in schizophrenia	Cohort	Portugal Outpatient center	Participants *n* = 9 Mean age = 38 Male = 7 Female = 2 Diagnosis = Schizophrenia	Self-help app CBT – 8 weeks. *No control*	Pre/post	PANSS General	*p* = 0.027 (*p* Wilcoxon) PANNS general psychopathology showed significance pre/post.	RAS ES GS-ES SSSS PSPS	*p* = 0.008 (*p* Wilcoxon) *p* = 0.017 *p* = 0.007 *p* = 0.021 *p* = 0.012 All other measures showed significance pre/post.
Bucci et al. (2018)	Actissist: Proof-of-concept trial of a theory-driven digital intervention for psychosis	RCT	United Kingdom Early intervention NHS	Participants *n* = 36 Age not reported. Male = 18 Female = 18 Diagnosis = Psychosis	Self-help app based on CBT – 12 weeks. CBT = 24 Control = 12	Pre/post & 22-week follow-up	PANSS Negative General Total PSYRATS	Cohen's *d*; 95% CI *PANSS negative* −0.85 (−1.58 to −0.12) *PANSS general* −0.86 (−1.44 to −0.25) *PANSS total* −0.85 (−1.44 to −0.25) No effect The PANSS general, negative symptoms and total showed a large effect pre/post. Results were not sustained at 22 week follow up.	CDSS GAF PSPS ERS EQ-5D-5L	Cohen's *d*; 95% CI −0.65 (−1.28 to −0.02) No effect No effect No effect No effect Only the CDSS total score showed a large effect pre/post. Results not sustained at 22-week follow up.
Granholm et al. (2012)	Mobile assessment and treatment for schizophrenia (MATS): a pilot trial of an interactive text-messaging intervention for medication adherence, socialization, and auditory hallucinations	Cohort	United States Outpatient treatment center	Participants *n* = 42 Mean Age = 48.70 Male = 29 Female = 13 Diagnosis = Schizophrenia or Schizoaffective	Text message intervention (guided) based on CBT– 12 weeks. *No control*	Pre/post	PANSS	No significant findings.	BDI-II ILSS	No significant findings.
Gottlieb et al. (2013)	Web-based cognitive-behavioral therapy for auditory hallucinations in persons with psychosis: A pilot study	Cohort	United States Outpatient mental health	Participants *n* = 21 Mean age = 40.1 Male = 13 Female = 8 Diagnosis = Schizophrenia or Schizoaffective	Self-guided CBT internet intervention – 10 weeks. *No control*	Pre/post	PSYRATS BAVQ-R	Auditory hallucination subscale *p* = 0.007 Not significant. PSYRATS significance pre/post intervention, auditory hallucination scale.	BDI-II BPRS	Not significant. *p* = 0.001 BPRS significance pre/post intervention.
Gottlieb et al. (2017)	Randomized controlled trial of an internet cognitive behavioral skills-based program for auditory hallucinations in persons with psychosis	RCT	United States Outpatientmental health	Participants *n* = 37 Mean age = 42.03 Male = 23 Female = 14 Diagnosis = Schizophrenia	Internet-based self-help CBT program – 10 weeks. CBT = 19 Control = 18	Pre/post & 3-month follow-up.	PSYRATS BAVQ-R PS	Not significant between groups. Not significant between groups. Not significant between groups.	BPRS BDI-II SLOF BCIS	Not significant between groups. Not significant between groups. *F*(1, 28) = 4.68, *p* = 0.039, ES = 0.43. Not significant between groups. SLOF significant between groups post intervention.
Hazell et al. (2018)	Guided self-help cognitive-behavior Intervention for VoicEs (GiVE): Results from a pilot randomized controlled trial in a transdiagnostic sample	RCT	United Kingdom NHS mental health outpatient	Participants *n* = 28 Mean Age = 42.50 Male = 11 Female = 16 Other = 1 Diagnosis = Psychosis	Guided self-help CBT – 12 weeks. CBT = 14 Control = 14	Pre/post (at 12 weeks)	HPSVQ	Cohen's *d*; 95% CI 1.78, (0.86–2.70 CI) Large significant effect found between groups on HPSVQ.	HADS SWEMBS RSES	Cohen's *d;* 95% CI 0.94, (0.13–1.75) 0.95, (0.13–1.75) 0.83 (0.03–1.63) Large significant effect for HADS, SWEMBS AND RSES between groups.
Moritz et al. (2016)	Effects of online intervention for depression on mood and positive symptoms in schizophrenia	RCT	Germany Mental health service	Participants *n* = 58 Mean Age = 40.80 Male = 27 Female = 31 Diagnosis = Schizophrenia or Schizoaffective	Internet CBT self-help– 12 weeks. CBT = 31 Control = 27	Pre/post (at 3 months)	The Paranoia Checklist PANSS	No significant group differences. No significant group differences.	PHQ-9 CES-D	*F*(1, 46) = 3.71, *p* = 0.06. Medium effect. *F*(1, 46) = 9.84, *p* = 0.003. Large effect. Significant difference between groups on CES-D with large effect. Significant different between groups on the PHQ-9 with a medium effect.
Naeem et al. (2016)	Cognitive behavior therapy for psychosis based guided self-help (CBTp-GSH) delivered by frontline mental health professionals: Results of a feasibility study	RCT	Canada Community-based treatment service	Participants *n* = 33 Mean age = 40.30 Male = 17 Female = 16 Diagnosis = Schizophrenia	CBT guided self-help – 12/16 sessions CBT = 18 Control = 15	Pre/post (at 16 weeks)	PANSS Positive Negative General PSYRATS Hallucinations Delusions	*F*(1, 30) = 6.77, *p* = 0.014. Cohen's *d* = 0.91. *F*(1, 30) = 7.35, *p* = 0.011. Cohen's *d* = 0.70. *F*(1, 30) = 6.68, *p* = 0.015. Cohen's *d* = 0.92. *F*(1, 30) = 13.18, *p* = 0.001 Cohen's *d* = 1.24. *F*(1, 30) = 7.47, *p* = 0.010. Cohen's *d* = 0.81. Significant and large effects found for PANSS and PSYRATS between groups post intervention.	WHODAS.20	*F*(1, 30) = 27.15, *p* = 0.000. Cohen's *d* = 1.99. Significant and large effect found for WHODAS.20 between groups post intervention.
Taylor et al. (2021)	A novel smartphone-based intervention targeting sleep difficulties in individuals experiencing psychosis: A feasibility and acceptability evaluation	Cohort	United Kingdom NHS community care team	Participants *n* = 14 Mean Age = 35.57 Male = 9 Female = 5 Diagnosis = Psychosis	Guided smart-phone CBT intervention – 6 weeks. *No control*	Pre/post	R-GPTS (Ideas of reference scale) SPEQ-H (Hallucination subscale)	Cohen's *d* = 0.49 (CI 1.25–7.48) Medium effect. No effect.	WSAS ISI PSQI DASS-21 Depression Anxiety Stress WEMWBS	Cohen's *d* = 0.27 (CI −0.32 to 6.32) Small effect. Cohen's *d* = 1.02 (CI 2.64–8.45) Large effect. Cohen's *d* = 0.83 (CI 0.91–5.64) Large effect. Cohen's *d* = 0.42 (CI 3.49–7.79) Small effect. Cohen's *d* = 0.35 (CI 95% −0.89 to 6.35) Small effect. Cohen's *d* = 0.24 (CI 95% −0.46 to 5.56) Small effect. Cohen's *d* = 0.26 (CI 95% −6.24 to −0.12) Small effect.
Westermann et al. (2020)	Internet-based self-help for Psychosis: Findings from a randomized controlled trial	RCT	Switzerland and Germany Community mental health center	Participants *n* = 101 Mean Age = 40 Male = 42 Female = 59 Diagnosis = Schizophrenia Spectrum Disorders	Guided internet self-help – 8 weeks CBT = 50 Control = 51	Pre/post (at 8 weeks) & 6-month follow-up	PANSS (positive factor) LSHS The paranoia checklist	*F*(1, 87.28) = 4.04, *p* = 0.047. Cohen's *d* = −0.37 (CI 95% −0.67 to −0.07) *F*(1, 88.22) = 7.15, *p* = 0.009. Cohen's *d* = 0.33 (CI −0.06 to 0.72). No significant findings. Follow up: positive effects and significance remained for the measures. No deterioration found.	WHOQOL	No significant findings.

*Note:* N/A, Not Applicable; RAS, Recovery Assessment Scale; ES, Empowerment scale; GS-ES, General Self-Efficacy Scale; SSSS, Social Support Satisfaction Scale; PSPS, Personal and Social Performance Scale; PANSS, Positive and Negative Syndromes Scale; PSYRATS, The Psychotic Symptom Rating Scale; CDSS, Calgary Depression Scale for Schizophrenia; GAF, Global Assessment of Functioning Scale; ERS, Empowerment Rating Scale; EQ-5D-5L, Health Status and Quality of Life; BDI-II, Beck Depression Inventory 2; ILSS, Independent Living Skills Survey; BAVQ-R, The Belief about Voices Questionnaire; BPRS, Brief Psychiatric Rating Scale; PS, Paranoia Scale; SLOF, The Specific Levels of Functioning Scale; BCIS, The Beck Cognitive Insight Scale; HPSVQ, Hamilton Program for Schizophrenia Voices Questionnaire (voice-impact subscale); WHODAS.20, WHO Disability Assessment Schedule; R-GPTS, Paranoid Thoughts Scale; SPEQ-H, Specific Psychotic Experiences Questionnaire; SPEQ-H (hallucination subscale), Specific Psychotic Experiences Questionnaire; WSAS, Work and Social Adjustment Scale; WEMWBS, Warwick-Edinburgh Mental Well-being Scale; DASS-21, Depression, Anxiety and Stress Scale; PSQI, Pittsburgh Sleep Quality Index; ISI, Insomnia Severity Index; LSHS, Launay-Slade Hallucination Scale; WHOQOL, Quality of Life Measure.

In regard to CBT-based self-help intervention, studies varied in terms of their treatment modality. Thus, four of the studies administered the CBT self-help intervention either using a mobile phone through a texting service, or through an App (Almeida et al., 2018; Bucci et al., 2018; Granholm et al., 2012; Taylor et al., 2021). Similarly, four studies utilized an internet-based intervention (Gottlieb et al., 2013, 2017; Moritz et al., 2016; Westermann et al., 2020) and the final two studies implemented standard written or paper-based self-help CBT treatments (Hazell et al., 2018; Naeem et al., 2016). From these interventions, they were either conducted unguided or guided by healthcare professionals. Five of the studies reported ‘guided’ interventions (Granholm et al., 2012; Hazell et al., 2018; Naeem et al., 2016; Taylor et al., 2021; Westermann et al., 2020). Five of the papers reported ‘unguided’ or ‘self-guided’ interventions (Almeida et al., 2018; Bucci et al., 2018; Gottlieb et al., 2013, 2017; Moritz et al., 2016). Interventions all ranged between six and twelve weeks in length. In order to measure the effectiveness of the CBT self-help interventions on psychotic symptoms, the most common outcome measure was the Positive and Negative Syndromes Scale (PANSS) which was used in six studies (Almeida et al., 2018; Bucci et al., 2018; Granholm et al., 2012; Moritz et al., 2016; Naeem et al., 2016; Westermann et al., 2020). All studies measured effectiveness using a pre-post design to measure treatment effectiveness, however three of these had follow-up periods ranging from 22 weeks to 6 months (Bucci et al., 2018; Gottlieb et al., 2017; Westermann et al., 2020).

### Study quality

Global ratings for quality appraisal can be found in Table 4. As presented, overall papers were rated as either weak (*n* = 5), moderate (*n* = 2) or strong (*n* = 3). Although high quality has not been achieved for all of the papers in this review, it is encouraging that half of the papers reported moderate to strong quality levels.

**Table 4.** Quality assessment scores – EPHPP quality assessment tool (Thomas et al., 2004)

Study	Selection bias	Study design	Confounders	Blinding	Data collection	Withdrawal and dropout	Global rating
Almeida et al. (2018)	Weak	Moderate	Weak	Moderate	Weak	Strong	Weak
Bucci et al. (2018)	Moderate	Strong	Weak	Weak	Weak	Strong	Weak
Granholm et al. (2012)	Moderate	Moderate	Weak	Moderate	Moderate	Strong	Moderate
Gottlieb et al. (2013)	Moderate	Moderate	Weak	Moderate	Weak	Strong	Weak
Gottlieb et al. (2017)	Moderate	Strong	Strong	Moderate	Moderate	Strong	Strong
Hazell et al. (2018)	Moderate	Strong	Weak	Moderate	Weak	Strong	Weak
Moritz et al. (2016)	Moderate	Strong	Strong	Strong	Strong	Strong	Strong
Naeem et al. (2016)	Moderate	Strong	Strong	Moderate	Strong	Strong	Strong
Taylor et al. (2021)	Moderate	Moderate	Weak	Moderate	Weak	Strong	Weak
Westermann et al. (2020)	Weak	Strong	Strong	Moderate	Strong	Moderate	Moderate

### Varieties of CBT-based self-help interventions

#### Guided and unguided interventions

Five of the studies used a form of guided self-help intervention (Granholm et al., 2012; Hazell et al., 2018; Naeem et al., 2016; Taylor et al., 2021; Westermann et al., 2020). Thus, Hazell et al. (2018) involved providing 1:1 support to participants whereby qualified clinical psychologists guided participants through the self-help workbook throughout the eight, one-hour long sessions. The study by Naeem et al. (2016) also included weekly support from health professionals, who guided participants through the self-help handouts and worksheets during the 1:1 therapy sessions. Similarly, Taylor et al. (2021) incorporated a trainee clinical psychologist into intervention delivery, who supported participants to complete the smartphone self-help intervention by providing a meeting before completion of the program, as well as offering 30-min contacts per week to trouble-shoot any technical difficulties, barriers to engagement and help to implement the CBT strategies. Comparatively, Westermann et al. (2020) implemented support to participants once a week by ‘guides’ who had at least a bachelor's degree in psychology. This support included checking through participants' online progress, provided written feedback and gave reminders to complete self-help tasks. Differing from the above studies, intervention guides within Granholm et al. (2012) provided daily support to participants, by sending 12 text-messages across six days in the week which involved delivery of the self-help intervention, in text message form.

In regard to the additional papers, the five final studies reported the use of ‘unguided’ means (Almeida et al., 2018; Bucci et al., 2018; Gottlieb et al., 2013, 2017; Moritz et al., 2016). For example, Gottlieb et al. (2013) involved study staff who provided general information on the self-help intervention and were available throughout to answer any questions. Moritz et al. (2016) provided video support throughout the entire intervention however, no personal feedback or direct therapeutic support was provided to participants. Both Almeida et al. (2018) and Gottlieb et al. (2017) provided information before completion of the therapy, on how to use the program. Finally, Bucci et al. (2018) involved video support within the intervention, explaining the therapy process. Weekly support was also given in order for participants to ask technical-based questions only.

#### Intervention platform

Appearing to reflect the shift towards remote therapies, two of the 10 papers included a face-to-face incorporated self-help CBT intervention (Hazell et al., 2018; Naeem et al., 2016). A variety of the interventions were conducted over the internet (Gottlieb et al., 2013, 2017; Moritz et al., 2016; Westermann et al., 2020). The most common intervention was conducted using a mobile phone, either through an App (Almeida et al., 2018; Granholm et al., 2012; Taylor et al., 2021) or alternatively using a text-messaging service (Bucci et al., 2018).

#### CBT intervention principles

All papers reported the use of a self-help intervention based on CBT principles. Two of the 10 studies cited models which were used as part of the CBT self-help intervention. For example, Hazell et al. (2018) involved a five-module intervention including topics of managing voices, targeting negative beliefs, targeting unhelpful beliefs, improving assertiveness and future planning of skills. Modules were based on the CBT model by Chadwick and Birchwood (1994), with the aim of reducing the impact of voices in people with psychosis. Similarly, Naeem et al. (2016) discussed using a CBT-based model for schizophrenia, originally developed by Turkington et al. (2008), which involved modules of psychoeducation, dealing with hallucinations, paranoia, challenging thoughts, behavioral activation, problem-solving and improving communication skills. Some studies developed their own CBT-based protocols. For example, Gottlieb et al. (2013, 2017) reported developing a CBT intervention with help from a clinical psychologist, an expert in CBT for psychosis. This intervention involved logging daily voice experience, rating distress, and program taught strategies to cope with the voice, video tutorials on psychosis and dysfunctional thinking, quizzes, and games to assist with applying concepts and practicing CBT coping skills. Strategies included self-monitoring, psychoeducation, cognitive distortions, and cognitive restructuring. Bucci et al. (2018) similarly reported developing a CBT intervention called ‘Actissist’ with the help from patients and key stakeholders, based on the cognitive model of psychosis. This intervention incorporated challenging unhelpful thoughts, providing alternative thinking, and using helpful coping strategies. Almeida et al. (2018) also designed and tested their own CBT intervention for schizophrenia within an MDT. Part of the intervention involved modifying patients' beliefs about delusions and hallucinations. Finally, Granholm et al. (2012) utilized a text messaging CBT intervention, ‘Mobile Assessment and Treatment for Schizophrenia’ MATS, which aimed to challenge unhelpful beliefs and incorporate the use of behavioral experiments. Both Moritz et al. (2016) and Taylor et al. (2021) incorporated interventions based on CBT frameworks. These typically included psychoeducation, thought challenging, and coping techniques for managing psychotic symptoms. Finally, Westermann et al. (2020) incorporated a CBT-based intervention for psychosis which involved the modules of paranoid ideation, voice hearing, self-esteem, sleep hygiene, metacognition, depression, mindfulness, worrying, social competence and relapse prevention.

### Effectiveness of CBT-based self-help interventions

All 10 studies explored the effectiveness of a form of CBT-based self-help intervention for psychotic symptoms. In addressing the second research question, this part of the narrative synthesis will focus on the effectiveness of the interventions in treating primarily psychotic symptoms, as well as other related symptoms.

### RCT study findings

#### Psychosis outcomes

The effectiveness of CBT-based self-help on psychotic related symptoms were explored within all six RCT's (Bucci et al., 2018; Gottlieb et al., 2017; Hazell et al., 2018; Moritz et al., 2016; Naeem et al., 2016; Westermann et al., 2020). Bucci et al. (2018) firstly explored pre-post psychotic outcomes using the PANSS. Immediate treatment effects 12 weeks post-intervention were found to be large on negative and general symptoms for psychotic symptomology compared to the control group. However, large effects were not sustained at 22-week follow-up. Similarly, Hazell et al. (2018) found large effects between groups on the HPSVQ, suggesting a large reduction in voice-hearing symptomology in psychotic presentations after completing the intervention post 12 weeks. Naeem et al. (2016) also reported reductions in psychotic symptoms post-intervention at 16 weeks compared to the control group on the PANSS and PSYRATS. Large treatment effects were noted for positive, negative, and general symptoms of psychosis. In addition, significantly large treatment effects were found on the PSYRATS hallucination and delusion scales. Finally, Westermann et al. (2020) found a significant small effect for post scores on the PANSS compared to the control group, as well as a small effect for the LSHS. Comparative to the studies which found an effect or significant finding, Gottlieb et al. (2017) found no significant differences pre- and post-intervention on psychotic symptomology on the PSYRATS, BAVQ-R or The Paranoia Scale. Moritz et al. (2016) also found there to be no effect on psychotic symptomology post-intervention on the PANSS and Paranoia Checklist compared to controls. Collectively, the majority of the studies report the benefits of CBT-based self-help on psychotic symptoms such as hallucinations and delusions; however, it is unclear whether effects are sustained over time due to lack of follow-up periods.

#### Secondary outcomes

Some psychosis-based studies also explored secondary outcomes, most of which were mental health related. Bucci et al. (2018) found there to be a large effect for the reduction of depressive symptoms on the CDSS post intervention compared to the control group. Similarly, Moritz et al. (2016) found there to be a large significant effect for depressive symptoms post-intervention compared to the control group on the CES-D, as well as a medium significant effect on the PHQ-9 measuring depression severity. Likewise, Hazell et al. (2018) reported large significant effects between pre- and post-intervention scores on depression, anxiety, self-esteem levels, and overall mental well-being. These findings suggest that individuals who experienced CBT-based self-help for psychosis had improved scores on depression, anxiety, self-esteem, and overall mental wellbeing.

Gottlieb et al. (2017) found a significant difference to occur between groups on a scale measuring daily functioning and daily living skills (SLOF) post-intervention. Similarly, Naeem et al. (2016) found there to be a large significant effect on pre-post scores on the WHODAS 2.0 for functioning and disability.

### Cohort study findings

#### Psychosis outcomes

The effectiveness of CBT-based self-help on psychotic related symptoms were explored within the four remaining cohort studies (Almeida et al., 2018; Gottlieb et al., 2013; Granholm et al., 2012; Taylor et al., 2021). Firstly, Almeida et al. (2018) reported significance on pre-post scores for the PANSS following an 8-week intervention, suggesting a high reduction in psychotic symptoms. Similarly, Gottlieb et al. (2013) reported a significant difference pre-post intervention on the PSYRATS, suggesting a significant reduction in auditory hallucination level after a 10-week self-help intervention. Finally, Taylor et al. (2021) reported a medium significant effect following intervention, on the R-GPTS, suggesting that paranoid thoughts significantly reduced following a period of intervention. In contrast to the majority of studies, Granholm et al. (2012) reported no significant findings in pre-post scores on the PANSS following the 12-week text-message-based CBT self-help intervention. Similar to the RCT studies, longitudinal effects are unclear due to lack of follow-up periods.

#### Secondary outcomes

Almeida et al. (2018) reported significant findings between pre and post-test scores on measures of recovery (RAS), empowerment (ES), self-efficacy (GS-ES), social support (SSSS) as well as personal and social performance (PSPS), suggesting that an improvement of symptoms for those with psychosis were also found within these additional areas. In addition, Gottlieb et al. (2013) reported significant findings post-intervention on the BPRS, a measure of general psychopathology, suggesting that scores significantly reduced following the intervention. Finally, Taylor et al. (2021) reported small to large effects to occur post intervention on measures assessing for work and social adjustment (WSAS), insomnia (ISI), sleep quality (PSQI), depression, anxiety, and stress (DASS-21) and overall wellbeing (WEMWBS).

## Discussion

50% of the papers investigated a form of ‘guided’ self-help whereby the intervention was supported by a facilitator (Granholm et al., 2012; Hazell et al., 2018; Naeem et al., 2016; Taylor et al., 2021; Westermann et al., 2020). The remaining papers explored ‘unguided’ self-help, independent of a facilitator (Almeida et al., 2018; Bucci et al., 2018; Gottlieb et al., 2013, 2017; Moritz et al., 2016). All studies were similar in the sense that no therapy support was provided in terms of therapeutic content. However, all studies provided a form of support to participants in regard to mostly technical aspects of accessing the intervention, as all unguided interventions were internet or mobile phone based. To summarize, it appears that both guided and unguided CBT-based self-help interventions have been developed for individuals with psychosis experience. In regard to intervention platform, most interventions were delivered either over the internet or through a mobile phone app (Almeida et al., 2018; Bucci et al., 2018; Gottlieb et al., 2013, 2017; Granholm et al., 2012; Moritz et al., 2016; Taylor et al., 2021; Westermann et al., 2020), compared to only two face-to-face examples of interventions (Hazell et al., 2018; Naeem et al., 2016). It appears that heavy weighting is towards self-help interventions using remote means, in comparison to face-to-face contact. Finally, all studies involved a self-help intervention based on CBT principles. Some studies incorporated a CBT intervention based on an existing model (Hazell et al., 2018; Naeem et al., 2016), with the aim of reducing psychotic symptoms such as hallucinations and delusions. The majority of the studies developed their own CBT protocol (Almeida et al., 2018; Bucci et al., 2018; Gottlieb et al., 2013, 2017; Granholm et al., 2012) with the similar aim of reducing psychotic symptoms through cognitive and behavioral strategies. Finally, the remaining two papers (Moritz et al., 2016; Taylor et al., 2021) involved CBT interventions based on some CBT principles such as psychoeducation and thought challenging.

### Psychosis outcomes

To summarize, it would appear that psychotic experiences significantly reduced following exposure to a self-help intervention based on CBT. Most notably, large significant treatment effects (Bucci et al., 2018; Hazell et al., 2018; Naeem et al., 2016), and small significant effects (Westermann et al., 2020) were evidenced across the majority of the RCT's.

In reducing psychotic symptoms, however the longevity of the effects remains unclear. The two remaining studies (Gottlieb et al., 2017; Moritz et al., 2016) reported no significant treatment benefits. A commonality between these two studies were their unguided means, which could tentatively pose questions as to whether unguided interventions are as effective as guided interventions. In view of the cohort studies, the majority of the studies reported significant findings on reducing psychotic experiences post-intervention (Almeida et al., 2018; Gottlieb et al., 2013; Taylor et al., 2021), however one study reported no significant benefit (Granholm et al., 2012). It therefore appears that most evidence suggests the benefits that CBT self-help can have in treating multiple symptoms of psychosis.

### Secondary outcomes

In view of the secondary outcomes, depression scores were commonly associated with significant treatment effect post intervention for psychosis (Bucci et al., 2018; Hazell et al., 2018; Moritz et al., 2016; Taylor et al., 2021), suggesting the benefit of CBT self-help in also reducing depressive symptoms in those with psychosis. Thus, tentative evidence was also found for the effectiveness of CBT self-help in various outcomes such as daily living skills and function (Gottlieb et al., 2017; Naeem et al., 2016), general psychological well-being (Gottlieb et al., 2013; Taylor et al., 2021) and anxiety (Hazell et al., 2018; Taylor et al., 2021). As the additional secondary outcomes varied greatly between each study, it is difficult to make firm conclusions, however it is clear that depression, daily living skills, general well-being and anxiety symptoms were most commonly found to improve post-intervention.

### Strengths and limitations

This current review addressed a gap in the literature and acknowledged potential research ideas suggested by Scott et al. (2015) in their previous review, whereby it was recommended that further research into CBT specific self-help for psychosis would be of relevance for clinical practice. This is therefore the first known review to explore solely the effectiveness of CBT-based self-help for psychosis, as well as providing further understanding of the varieties of CBT self-help interventions available for those experiencing psychosis. Various strengths can be identified in the review. Thus, a systematic approach abiding by a thorough PRISMA checklist was utilized in this review, whereby each stage of the process has been made transparent and concise for the reader. There are also some limitations which are important to acknowledge. Firstly, the majority of the studies were either rated as ‘weak’ (*n* = 5) or ‘moderate’ (*n* = 2) in quality which can impact on reliability of the study findings. More so, grey literature was excluded from this review due to the lack of peer-reviewed processes (Paez, 2017). Therefore, niche, or emerging research findings may have been overlooked, which may have impacted the results. Furthermore, a variety of the papers involved in the review were a cohort design. Within these, the self-help CBT intervention was not compared to a control group, therefore creating difficulty in drawing meaningful assumptions regarding the effectiveness of the intervention, with lack of control for confounding variables. In addition, most of the studies did not include a follow-up after the intervention period. Of these, only three studies completed follow-ups between 22-weeks and six months. This makes it difficult to draw firm conclusions on the effectiveness of the self-help interventions over a longer period of time.

With regard to the limitations discussed within this review, there are several areas for direction of future research. As a large proportion of the studies were cohort in nature with the lack of a control group, future studies could focus on continuing to investigate the effectiveness of CBT-based self-help for psychosis within randomized controlled designs to increase methodological rigor. To address issues with lack of follow-ups, future studies could also ensure follow-up periods are included within their study design, to ascertain effectiveness of the interventions over time. Additionally, due to significant issues with quality ratings for the majority of the studies, further research could also focus on addressing methodological difficulties, creating high-quality research to review. A meta-analysis was not performed due to significant study heterogeneity (e.g. differences in outcome measures and samples) and issues with study quality. Thus, a replication of the current review once supplementary high-quality research has been completed would be advantageous, with the inclusion of a meta-analysis. This would enable more confidence to be drawn from this review, investigating the effectiveness of CBT-based self-help interventions for psychosis.

## Conclusion

After weighing up the findings, this review provides credible evidence for the short-term effectiveness of CBT-based self-help for reducing symptoms of psychosis, whereby seven studies concluded the effectiveness in reducing psychotic symptomology post-treatment, however the longevity of effectiveness remains unclear. Some support has also been found for secondary outcomes such as depression, overall well-being, daily functioning, and anxiety across a small variety of studies, however additional research is needed to gain further certainty with these effects.

## Supporting information

Kruger et al. supplementary material Kruger et al. supplementary material

## Supplementary material

The supplementary material for this article can be found at https://doi.org/10.1017/S0033291724001545.
